# Clinical Efficacy of Chlorhexidine Gargle Combined with Recombinant Bovine Basic Fibroblast Growth Factor Gel in the Treatment of Recurrent Oral Ulcers and Its Effects on Inflammatory Factors, Immune Function, and Recurrence Rate

**DOI:** 10.3290/j.ohpd.b5081283

**Published:** 2024-03-14

**Authors:** Haishan Cui, Pinghua Wang, Meiling Chen, Shanshan Lu

**Affiliations:** a Attending Physician, Department of Stomatology, First People’s Hospital of Chun’an County, Hangzhou, Zhejiang, China. Study design, edited the manuscript, read and approved the final version of the manuscript.; b Attending Physician, Department of Stomatology, First People’s Hospital of Chun’an County, Hangzhou, Zhejiang, China. Performed experiments, read and approved the final version of the manuscript.; c Attending Physician, Department of Stomatology, First People’s Hospital of Chun’an County, Hangzhou, Zhejiang, China. Data analysis, read and approved the final version of the manuscript.; d Attending Physician, Department of Stomatology, Nanjing Liuhe Traditional Chinese Medicine Hospital, Nanjing, Jiangsu, China. Edited the manuscript, read and approved the final version of the manuscript.

**Keywords:** chlorhexidine gargle, clinical efficacy, immune function, inflammatory factors, recombinant bovine basic fibroblast growth factor gel, recurrence rate, recurrent oral ulcers

## Abstract

**Purpose:**

To examine the clinical efficacy of a chlorhexidine gargle combined with recombinant bovine basic fibroblast growth factor (rb-bFGF) gel in the treatment of recurrent oral ulcers and its effects on inflammatory factors, immune function, and recurrence rate.

**Materials and Methods:**

Ninety-six patients with recurrent oral ulcers were randomly assigned to two groups: experimental (treatment with chlorhexidine gargle plus rb-bFGF gel) and control (treatment with chlorhexidine gargle alone) (n = 48 cases). The therapeutic efficacy, clinical improvement of symptoms, and recurrence rate within 3 months were compared between the two groups. Serum inflammatory factor and immune factor levels of patients in the two groups were measured before and after treatment.

**Results:**

A statistically significantly higher total effective rate was found in patients of the experimental group (95.83%) versus the control group (81.25%) (p < 0.05). The time to onset of pain relief was shortened, the duration of pain relief was prolonged, and VAS scores for pain level were lower in the experimental than the control group (p < 0.05). Among patients in the experimental group, the number of oral ulcers and ulcer area decreased, and faster onset of pain relief and time until normal eating improved in comparison to the control group (p < 0.05). Reduced levels of IL-2, IL-6, IL-8, and TNF-α were observed in the experimental vs the control group (p < 0.05). Elevated levels of CD3+, CD4+, and NKT and reduced levels of CD8+ were found in the experimental group compared to the control group (p < 0.05). The ulcer recurrence rate of patients in the experimental group (8.33%) was notably lower in comparison to the control group (29.17%).

**Conclusion:**

Chlorhexidine gargle plus rb-bFGF gel can improve the clinical outcome of patients with recurrent oral ulcers. It can reduce the levels of inflammatory factors, improve immune function, and reduce the recurrence rate.

Recurrent oral ulcers represent a group of diseases characterised by recurrent episodes of benign, infectious or noninfectious, and spontaneously-healing ulcers in healthy individuals.^22^ Immunological factors, inflammatory cascade, infectious agents, as well as systemic conditions, are the chief reasons for the expansion of oral ulcers, and oxidative stress after activation of the inflammatory cascade functions in the pathogenesis of oral ulcers.^9^ Oral ulcers are usually treated at home without professional care, and because recurrent oral ulcers are generally self-healing, people often tend to tolerate them or use alternative treatments instead of seeking professional advice.^28^ Many chemical and biological agents have been suggested to be beneficial in treating patients with recurrent oral ulcers, but to date no definitive treatment exists.^6^ Compounded by their periodicity, recurrent oral ulcers are extremely painful, which seriously impacts the patient’s quality of life.^11^

Chlorhexidine gargle (or mouth rinse) is a commonly used anti-inflammatory medication containing glycerin, metronidazole, concentrated mint solution, and chlorhexidine gluconate, which is highly effective in inactivating *Peptococcus* spp., *Candida* *albicans*, and gram-negative/positive bacteria. Because the rinse is held in the mouth, it can fully contact the ulcer surface and germicidally act directly on the affected area. Chlorhexidine is a frequently prescribed antiseptic agent in the field of dentistry. It has long-lasting antibacterial efficacy with a wide range of action, and decreases plaque, gingival inflammation, and bleeding.^32^ Chlorhexidine is a base that is as stable as salt. Chlorhexidine gluconate, as the most frequent oral preparation, is water soluble, easily dissociates, and releases the positively charged chlorhexidine ingredient at a physiological pH of 10. When administered as chlorhexidine gluconate, dilution with saliva and the cleaning of the oral mucosa decrease its bioavailability, which compromises its efficacy.^8^ Metronidazole, a derivative of nitroimidazole, is a distinct antimicrobial agent active against bacteria and parasitic micro-organisms.^25^ It is also used for the treatment of some types of infections, while coexisting conditions or concomitant use with other drugs may hinder its efficacy.^30^ Fibroblast growth factor (FGF) is a class of secreted polypeptide ligands broadly utilised for treating burns, chronic and fresh wounds, and repair of corneal lesions.^12,18^ Basic FGF (bFGF) has good angiogenesis and ulcer-healing effects, which can trigger neural regeneration in the central nervous system.^20^ Recombinant bovine bFGF (rb-bFGF) gel can effectively reduce atrophic acne scars and mitigate local inflammatory reactions, with good efficacy and few adverse reactions.^7^ In addition, rb-bFGF has been suggested to enhance the proliferation, repair, and regeneration of ectodermal, neuroectodermal, and mesoderm-derived cells, boost capillary regeneration, and improve both local circulation and wound healing.^24,36^ In this study we aimed to examine the clinical efficacy of chlorhexidine gargle combined with rb-bFGF gel in the treatment of recurrent oral ulcers and its effects on inflammatory factors, immune function, and recurrence rate.

## Materials and Methods

The research was approved by the Ethics Committee of our hospital, and the patients supplied written informed consent.

### Participants

Ninety-six patients with recurrent oral ulcers admitted to the Stomatology Department of our hospital were chosen and randomly assigned to an experimental group and a control group following randomised numerical table methods (n = 48 cases). Patients in the control group were treated with chlorhexidine gargle alone (South China Pharmaceutical; Shenzhen, China), and those in the experimental group were treated with rb-bFGF gel (EssexBio, Zhuhai, China) plus chlorhexidine gargle.

The inclusion criteria were as follows: 1. Patients met the diagnostic criteria of recurrent oral ulcer (Guidelines for the Diagnosis and Treatment of Recurrent Oral Ulcer [trial]); 2. patients age was ≥ 18 years; 3. patients had a course of disease ≥ 6 months and a frequency of recurrence ≥ 1 time/month; 4. no ulcers or erosions were present in other parts of the body; 5. no local irritants existed in the oral cavity; 6. the patients all volunteered to participate in this trial and signed an informed consent form. Patients were excluded if they: 1. had non-recurrent oral ulcers, such as traumatic ulcers, leukoaraiosis, and other ulcers; 2. intended to become pregnant, were pregnant or breastfeeding; 3. had allergies or hypersensitivity to the drugs used in this trial; 4. presented with multi-systemic diseases or psychiatric disorders, or were unable to understand and cooperate with the trial; 5. had taken antibiotics or immunologically or hormonally active drugs in the last month.

The control group consisted of 19 males and 29 females with a mean age of 30.58 ± 4.10 years and a mean disease duration of 2.79 ± 0.50 years. The experimental group comprised 31 females and 17 males with a mean age of 31.27 ± 4.36 years and a mean disease duration of 2.71 ± 0.58 years. The difference in general information between the two groups was not statistically significant (p > 0.05) and was comparable.

### Treatments

Patients in the experimental and control groups underwent dietary management, were instructed not to eat spicy and stimulating foods, and were administered vitamin B tablets (Reyoung Pharmaceutical; Shandong, China) as follows: 2 tablets 3 times per day.

In the control group, participants used chlorhexidine gargle (South China Pharmaceutical; Shenzhen, China) for 5 min after brushing their teeth in the morning and evening, that is, 2 × 10 ml/day.

Patients in the experimental group were given rb-bFGF gel plus chlorhexidine gargle 4 times/day. Patients were instructed to rinse their mouths using dilute saline and maintain oral hygiene before using rb-bFGF gel. After the mouth ulcers were exposed and dried, the gel was gently applied to the affected area, and food and water were prohibited for 30 min after application of gel in order to facilitate its full absorption. The treatment course for both groups of patients was 1 week.

### Clinical Efficacy

The clinical efficacy noted in the two groups of patients was rated markedly effective, effective, or ineffective. The disappearance of pain and other clinical symptoms along with the healing of ulcers after 3 days of treatment was regarded as markedly effective. An obvious reduction of pain and other clinical symptoms along with the healing of ulcers after 5 days of treatment was regarded as effective. A lack of pain reduction, no improvement in other clinical symptoms, and no healing of ulcers after 7 days of treatment was regarded as ineffective. The total effective rate was calculated as (number of markedly effective cases + number of effective cases)/total number of cases x 100%.

### Measuring Ulcer-related Pain

The pain level of the two groups of patients before and after treatment was compared using a 10-point visual analog scale (VAS), in which a low score indicated a low pain level. The pain relief onset time and duration of pain relief of the two groups of patients were also recorded.

### Healing of Oral Ulcers

The healing of oral ulcers was assessed based on the number of oral ulcers, ulcer area, time until onset of pain relief, and time until normal eating improved in the two groups of patients after treatment.

### Serum Inflammatory Factor Levels

Elbow-vein blood (3 ml) was drawn from patients before and after treatment. The serum was separated via centrifugation and stored at -80°C in a freezer until testing. Serum IL-2, IL-6, IL-8, and TNF-α levels were detected using ELISA kits from R&D Systems (Minneapolis, MN, USA) following the manufacturer’s recommendations and using a microplate reader SpectraMax I3 (Molecular Devices; San Jose, CA, USA). The inflammatory response of patients was assessed by comparing the levels of inflammatory factors before and after treatment in the two groups.

### Serum Immune Factor Levels

Early morning fasting venous blood (3 ml) was drawn from patients before and after treatment, and the serum was obtained by centrifugation. T-cell subpopulations (CD3+, CD4+, and CD8+) and natural killer T-cell (NKT) levels were examined using a CytoFLEX flow cytometer (Beckman Coulter; Suzhou, China). The immune response of patients was evaluated by comparing the changes in the levels of immune factors in the two groups.

### Disease Recurrence

All patients were followed up for 3 months after treatment. The recurrence of clinical symptoms was considered recurrence. The recurrence rate was calculated and compared between groups.

### Statistical Methods

SPSS 20 (IBM; Armonk, NY, USA) and GraphPad Prism 6.0 software (Graph Pad; La Jolla, CA, USA) were applied to process the data. Measurement data were expressed as mean ± standard deviation and categorical data were expressed as %. If the tests were consistent with normal distribution and homogeneity of variance, two-group comparisons of the data were performed using the t-test. Categorical data were analysed using the *χ*^2^ test. p < 0.05 was regarded as a statistically significant.

## Results

### Clinical Efficacy

A statistically significantly higher total effective rate was found in patients of the experimental group (95.83%) vs the control group (81.25%) (p < 0.05; Table 1). In short, chlorhexidine gargle plus rb-bFGF gel had greater clinical efficacy (experimental group) than did chlorhexidine alone (control group).

**Table 1:** Comparison of clinical efficacy between the experimental and control groups

Group	Marked effective	Effective	Ineffective	Total effective rate
Control group (n = 48)	20 (41.67%)	19 (39.58%)	9 (18.75%)	39 (81.25%)
Experimental group (n = 48)	30 (62.50%)	16 (33.33%)	2 (4.17%)	46 (95.83%)
p-value				0.025

### Ulcer Pain

Compared to the control group, a shorter time to onset of pain relief and a longer duration of pain relief were evident in the experimental group (p < 0.05). Reduced VAS scores were noted in patients of both groups after treatment vs before treatment (p < 0.05), and the experimental group exhibited a further reduction (p < 0.05) (Table 2). It is suggested that treatment with chlorhexidine gargle alone as well as with chlorhexidine gargle plus rb-bFGF gel statistically significantly mitigates patients’ ulcer pain, but the combination of drugs is more effective.

**Table 2:** Comparison of ulcer pain between the experimental and control groups

Group	Pain relief onset time (min)	Duration of pain relief (min)	VAS (point)	
			Before treatment	After treatment
Control group (n = 48)	6.92 ± 1.17	20.40 ± 3.43	6.06 ± 1.11	3.13 ± 0.72*
Experimental group (n = 48)	2.90 ± 0.77	38.69 ± 4.51	6.38 ± 1.07	1.06 ± 0.24*
p-value	<0.001	<0.001	0.154	<0.001

*p < 0.05 compared to before treatment within the same group.

### Healing of Oral Ulcers

After treatment, the number of oral ulcers and ulcer area had decreased, onset of pain relief was faster and the time until normal eating improved was shorter in patients in the experimental group vs the control group (p < 0.05) (Table 3). That is, chlorhexidine gargle plus rb-bFGF gel better promoted the healing of oral ulcers than did chlorhexidine gargle alone.

**Table 3:** Comparison of healing of oral ulcers between the experimental and control groups

Group	Number of oral ulcers	Ulcer area (mm^2^)	Onset of pain relief (days)	Time until normal eating improved (days)
Control group (n = 48)	2.79 ± 0.71	1.73 ± 0.51	5.23 ± 1.07	5.29 ± 1.14
Experimental group (n = 48)	0.90 ± 0.31	0.49 ± 0.18	2.50 ± 0.84	2.81 ± 0.95
p-value	<0.001	<0.001	<0.001	<0.001

### Serum Inflammatory Factor Levels

There was no statistically significant difference in inflammatory factors (IL-2, IL-6, IL-8, and TNF-α) between the two groups before treatment (p > 0.05). Reduced levels of IL-2, IL-6, IL-8, and TNF-α were observed in both groups after treatment vs before treatment in the same group, and the levels of these parameters in the experimental group were statistically significantly lower than in the control group (p < 0.05; Table 4). Thus, chlorhexidine gargle plus rb-bFGF gel hindered the inflammatory reaction.

**Table 4:** Comparison of serum inflammatory factor levels between the experimental and control groups

Group	IL-2 (ng/L)		IL-6 (ng/L)		IL-8 (ng/L)		TNF-α (ng/L)	
	Before treatment	After treatment	Before treatment	After treatment	Before treatment	After treatment	Before treatment	After treatment
Control group (n = 48)	37.91 ± 5.76	29.28 ± 6.13*	10.83 ± 2.15	8.24 ± 1.02*	19.83 ± 3.77	14.62 ± 3.24*	26.83 ± 3.53	18.42 ± 2.27*
Experimental group (n = 48)	39.02 ± 5.55	23.46 ± 6.56*	11.14 ± 2.24	5.24 ± 0.95*	18.85 ± 3.70	11.03 ± 2.73*	27.05 ± 3.27	12.31 ± 2.12*
p-value	0.339	<0.001	0.491	<0.001	0.202	<0.001	0.752	<0.001

*p < 0.05 compared to before treatment within the same group.

### Serum Immune Factor Levels

No statistically significant difference was observed in immune factors (CD3+, CD4+, CD8+, and NKT) between the two groups before treatment (p > 0.05). Elevated levels of CD3+, CD4+, and NKT and reduced levels of CD8+ were noted in both groups after treatment vs before treatment in the same group (p < 0.05). Elevated levels of CD3+, CD4+, and NKT and reduced levels of CD8+ were found in the experimental group vs the control group (p < 0.05; Table 5). This demonstrates that chlorhexidine gargle plus rb-bFGF gel activates immune cells and regulates the immune system.

**Table 5:** Comparison of serum immune factor levels between the experimental and control groups

Group	CD3+ (%)		CD4+ (%)		CD8+ (%)		NKT (%)	
	Before treatment	After treatment	Before treatment	After treatment	Before treatment	After treatment	Before treatment	After treatment
Control group (n = 48)	52.04 ± 6.76	64.39 ± 8.08*	24.83 ± 4.01	32.27 ± 5.25*	35.75 ± 4.04	26.64 ± 3.73*	4.18 ± 1.11	5.05 ± 1.45*
Experimental group (n = 48)	51.79 ± 6.55	73.85 ± 8.73*	25.17 ± 4.44	39.74 ± 5.96*	34.94 ± 4.72	23.08 ± 3.30*	4.06 ± 1.02	7.72 ± 2.03*
P value	0.854	<0.001	0.695	<0.001	0.369	<0.001	0.583	<0.001

*p < 0.05 compared to before treatment within the same group.

### Recurrence Rate of Oral Ulcers

After following-up the patients for 3 months post-treatment, we found that 14 patients in the control group and 4 patients in the experimental group experienced recurrence. The ulcer recurrence rate in the experimental group (8.33%) was statistically significantly lower than in the control group (29.17%) (p < 0.05).

## Discussion

A recurrent oral ulcer is a common disease that seriously impacts the quality of life, and is usually linked to systemic diseases, including Crohn’s disease, Behçet’s disease, and ulcerative colitis.^26^ Because of the uncertain causes and unpredictable disease course, the clinical treatment of recurrent oral ulcer mainly focuses on pain relief, prolonging the intermission period, as well as shortening the course of disease.^6^ In this study, we aimed to determine the clinical efficacy of the chlorhexidine gargle combined with rb-bFGF gel in the treatment of recurrent oral ulcers and its effects on inflammatory factors, immune function, and recurrence rate. Ninety-six patients with recurrent oral ulcers were selected and randomly placed into either the experimental group (chlorhexidine gargle plus rb-bFGF gel) or the control group (chlorhexidine gargle alone). The therapeutic efficacy, symptom improvement, and recurrence rate within 3 months of patients in the two groups were compared. Serum inflammatory factor and immune factor levels of patients in the two groups were measured before and after treatment.

The chlorhexidine gargle used here (South China Pharmaceutical; Shenzhen, China) is mainly composed of chlorhexidine and metronidazole. Chlorhexidine is widely utilised as an antimicrobial, antiseptic, and anti-plaque agent, and is available as topical gels, mouthrinses, antiseptic skin creams, and also disinfectant for the preparation of the skin before surgery.^23^ In the oral cavity, the proposed mechanism of chlorhexidine involves reducing pellicle formation, changing bacterial adherence to the teeth, and changing the permeability of bacterial cell walls, which ultimately contributing to cell lysis.^19^ Metronidazole is the chemotherapy drug of choice for necrotising ulcerative gingivitis, and it is applied in high-dose chemoprevention and for the therapy of many strictly anaerobic infections.^2^ The systemic administration of metronidazole has been found to contribute to notable additional improvements in radiographic, clinical, as well as microbiological parameters after 12 months of follow-up in treating peri-implantitis.^3^ Furthermore, metronidazole-assisted scaling and root planing, along with personal oral hygiene guidance, can significantly improve the health of the periodontium.^33^ The articles cited above verify the treatment effect of chlorhexidine and metronidazole (the main components of the chlorhexidine gargle) in oral-cavity-related diseases. The emergence of resistance to all types of antibiotics among previously susceptible bacterial pathogens poses the main challenge to infectious-disease medicine, as the overuse of antibiotics contributes to an increase in bacterial resistance.^13^ Over the last few decades, numerous reports have demonstrated an elevation in bacterial resistance against chlorhexidine in diverse species.^4,5^ Nevertheless, there is no clear finding that provides a basis for universal clinical practice. Besides, the cross-resistance to chlorhexidine with other antibiotics and biocides has been reported, revealing that the mechanism of cross-resistance is supported by the elevated resilience of the outer membrane of chlorhexidine-resistant strains.^1^ Nevertheless, with the widespread exposure to chlorhexidine, reports of adverse reactions are increasing. Its complications range from mildly irritating contact dermatitis to life-threatening anaphylaxis.^27^ Meanwhile, reversible local adverse effects, such as impaired taste sensation, staining of teeth, dental restorations, discoloration of the tongue, elevated formation of supragingival calculus, along with occasional mucous membrane irritation and desquamation, are related to the prolonged use of CHX mouthrinse.^31^

bFGF participates in granulation tissue formation and angiogenesis, increasing the blood supply to the wound area, and functions in its healing.^17^ More specifically, Kibe et al^14^ have shown that bFGF is implicated in keratinocyte proliferation, differentiation, and migration into scar areas, leading to granulation tissue neovascularisation in wound healing. A study by Ohshima et al^22^ found that bFGF exerts a healing effect on oral ulcers and simultaneously promotes the wound healing process, causing granulation formation and promoting ulcer healing. Other authors reported that bFGF can enhance angiogenesis in the oral mucosa and accelerate wound healing.^35^ A significant beneficial influence of topical use of rb-bFGF gel has been found in patients with facial dermatitis.^16^ The application of rb-bFGF has been demonstrated to reduce erythema, desquamation, papules, and dryness; it is particularly useful in treating rosacea granuloma lesions and in preventing late scar formation.^15^ These findings suggest a positive treatment effect of rb-bFGF gel in oral-cavity-related diseases.

The present findings demonstrate that chlorhexidine gargle plus rb-bFGF gel could improve the clinical outcome of patients with recurrent oral ulcers. The reason may be that the combination of the two drugs can activate immune cells and regulate the body’s immune system, thereby promoting wound healing. As previously reported, chlorhexidine gluconate or turmeric gel can be effective in the prevention of plaque and gingivitis, and chlorhexidine gluconate gel is more effective than turmeric gel in terms of anti-plaque and anti-inflammatory capabilities.^ 29^ In a study on treating secondary cicatricial alopecia, Yang et al^35^ found that follicle-unit extraction transplantation plus rb-bFGF and minoxidil can decrease the rate of hair loss and enhance both hair survival and satisfaction rates, with fewer adverse reactions compared with follicle unit extraction transplantation alone.^4^

## Conclusion

This study shows that chlorhexidine gargle plus rb-bFGF gel can improve the clinical outcome in patients with recurrent oral ulcers, reduce the levels of inflammatory factors, improve immune function, and reduce the recurrence rate. The use of the chlorhexidine gargle plus rb-bFGF gel in treating oral ulcers is clinically effective and should be widely promoted. However, due to the limited sample size in this study and the short study period, the clinical effectiveness of chlorhexidine gargle plus rb-bFGF gel in the treatment of recurrent oral ulcers needs to be confirmed by further trials with larger sample sizes.
